# Supplementary material for: Spatial confinement affects the heterogeneity and interactions between shoaling fish
Source: Sci Rep. 2024 May 29;14:12296. doi: 10.1038/s41598-024-63245-y (PMC11711749; doi:10.1038/s41598-024-63245-y)
Supplement: Supplementary file 1 — Supplementary Information. [file 41598_2024_63245_MOESM1_ESM.pdf]

Supplement to: Spatial Confinement Affects the Heterogeneity and Interactions Between Shoaling Fish

Gabriel Kuntz[a], Junxiang Huang[b], Mitchell Rask[a], Alex Lindgren-Ruby[a], Jacob Y. Shinsato[a], Dapeng Bi[b], and A. Pasha Tabatabai[a,c,*]

[a]Seattle University, Department of Physics, Seattle WA, 98122, USA

[b]Northeastern University, Department of Physics, Boston, MA, 02115, USA

[c]California Polytechnic State University, Physics Department, San Luis Obispo CA, 93407, USA

[*]pashatab@calpoly.edu

April 10, 2024

1 Fitting with the k-gamma Distribution

This robust fit single parameter fit around the modal area A_0 supplements the analysis of the parameters k_c and k_e as delineated in Main Text Equation 3.

Let $A_0 = (k-1)\theta$ denote the mode of the Gamma distribution, and ΔA represent a deviation from this mode. Main Text Equation 3 values at $A = A_0 \pm \Delta A$ becomes

$$f_\Gamma(A_0) - f_\Gamma(A_0 \pm \Delta A) = \frac{e^{-(k-1)}[(k-1)\theta]^{k-1}}{\theta^k \Gamma(k)}[1 - (1 \pm \frac{\Delta A}{A_0})^{k-1} e^{\mp \frac{(k-1)\Delta A}{A_0}}]. \quad (1)$$

We use $\Delta A = (\frac{1}{2} \pm \frac{1}{6})A_0$ to estimate k_e and k_c. Consequently, the ratio k_e/k_c can be derived as follows:

$$k_e/k_c = \frac{f_\Gamma(A_0) - f_\Gamma(A_0 + \frac{A_0}{2})}{f_\Gamma(A_0) - f_\Gamma(A_0 - \frac{A_0}{2})} = \frac{1 - (\frac{3}{2})^{k-1} e^{-\frac{k-1}{2}}}{1 - (\frac{1}{2})^{k-1} e^{\frac{k-1}{2}}}, \quad (2)$$

which solely depends on k. We list the calculated k_e/k_c ratio values in Table 1.

2 Supplementary Figures

‘

R(cm)	k	k_e/k_c
8	11	0.72
11	8.3	0.66
14	5.3	0.59
34.25	4.2	0.57
44.25	2.2	0.52

Supplementary Table 1: The k_e/k_c ratio calculated from Equation (2) as a function of radius R.

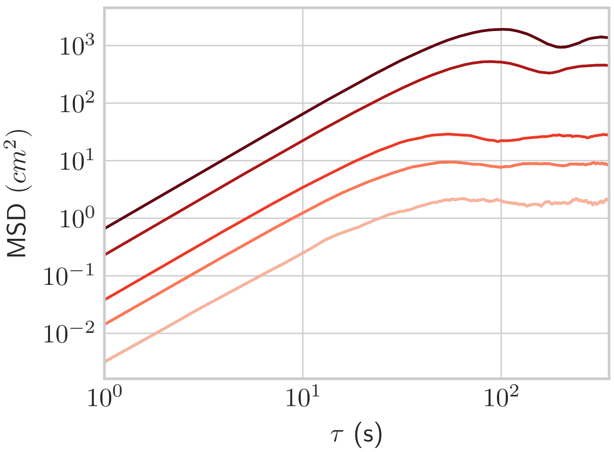

Supplementary Figure 1: Mean Squared displacement for the center of mass of 25 fish in the different arenas. As Arena radii decreases color lightens.

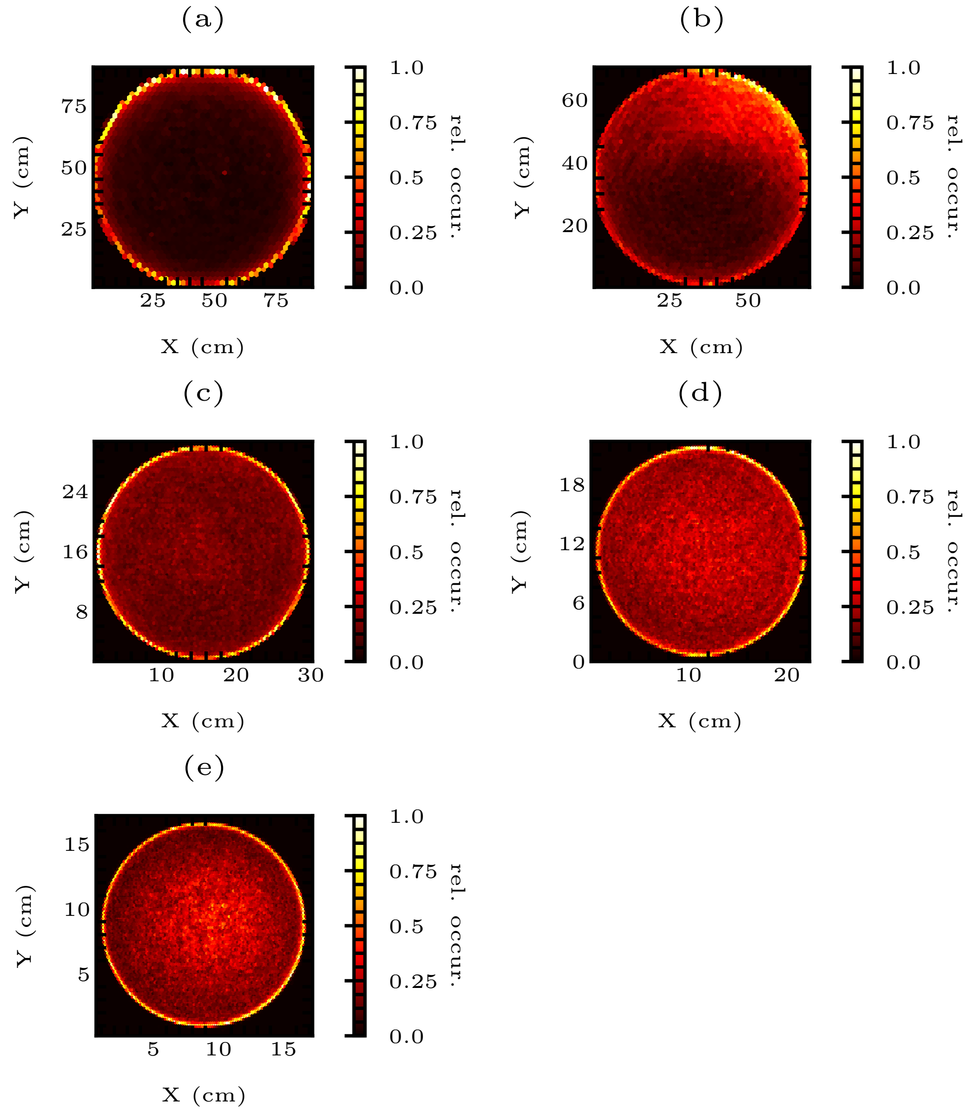

Supplementary Figure 2: Relative occurrences of fish positions accumulated over a single experiment (25 minutes). Color bars are normalized to spatial bin with largest number of counts. Panels a-e are representative images for arenas of different radii (R). All containers have 25 fish. (a) $R = 44.45$cm Arena, 322564 total occurrences (b) $R = 34.25$cm Arena, 337650 total occurrences (c) $R = 14.3$cm Arena, 335019 total occurrences (d) $R = 11$cm Arena, 347861 total occurrences (e) $R = 8$cm Arena, 343816 total occurrences

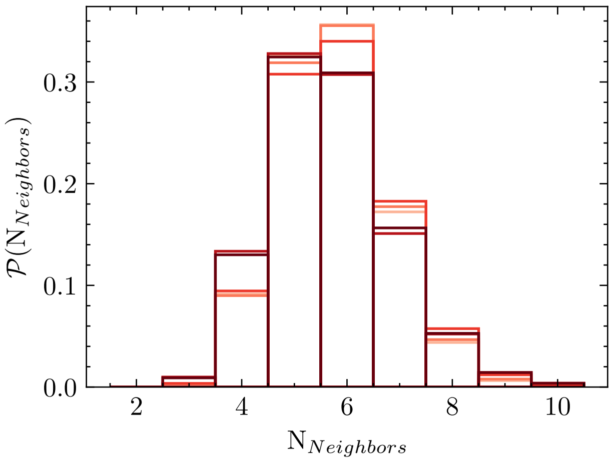

Supplementary Figure 3: Probability distribution of the number of topological neighbors of inside fish grouped by arena size. Colors darken with increasing R with R = 8cm, 11 cm, 14cm, 34.25cm and 44.45cm with average $\mathrm{N}_{Neighbors}$: 5.76, 5.79, 5.83, 5.70, and 5.72, respectively.

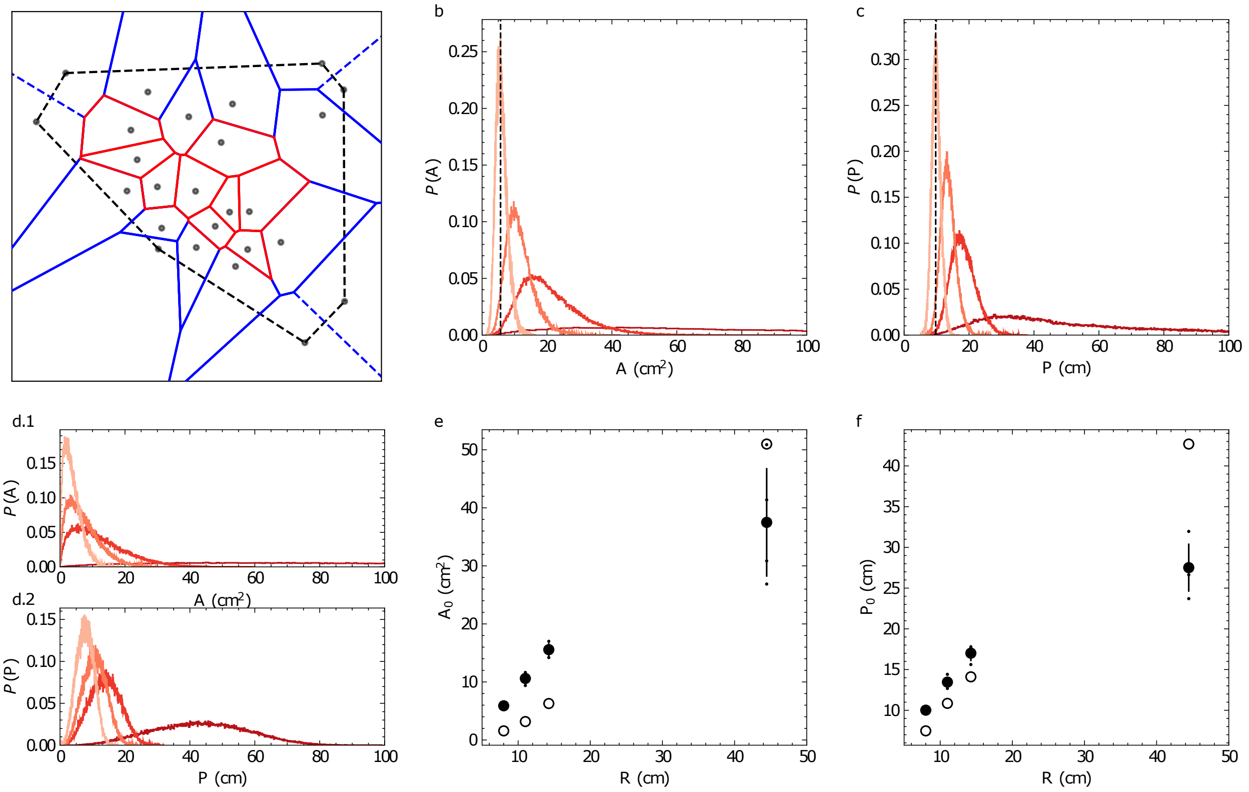

Supplementary Figure 4: (a) Voronoi tessellation (blue polygons) of 25 fish. Fish positions are grey markers. Convex hull is defined by black dashed line. Internal fish (red polygons) have all vertices within convex hull. Image has length 90cm. (b) Probability distributions of internal areas (A) for various arena sizes. (c) Probability distribution of internal area perimeters (P) (d) Probability distributions of (d.1) areas (A) and (d.2) perimeters (P) for 25 randomly generated points in simulated arenas. (b),(c), and (d) arena areas in radii R = 8cm, 11 cm, 14.25cm and 44.45cm as shade darkens respectively. (e)(f) Peaks of area (perimeter) distributions A_0 (P_0) as a function of arena radii (R). Each data point is a small black point and the average (Large Black Point) is plotted with a 1 STD error bar for each arena size (R). Peaks of area (perimeter) distributions for randomly placed points (open symbols). (Put entirity of this figure version into supplement. replace with one -P -random +hull +neighbors)

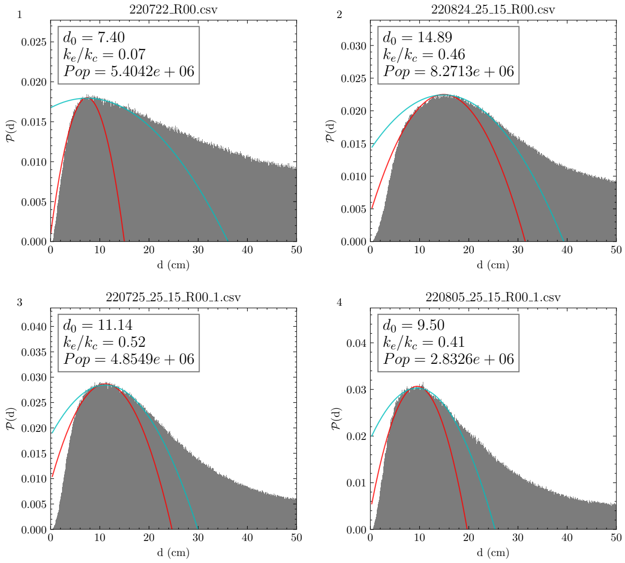

Supplementary Figure 5: Probability distributions of distances (d) between all fish in a frame for four separate replicate experiments (1-4) with 25 fish in the largest arena ($R = 45.44$cm). Distributions are fit with two parabolas centered around the most occurring value (d_0). The ratio of the coefficients for the parabolas (k_e/k_c) and population number of data points (Pop) in each probability distribution is included. The fit for 4 is centered around a point that better reflects the peak of the curve and not the most occurring bin. The most occurring bin for 4 has a distance value of 10.74.

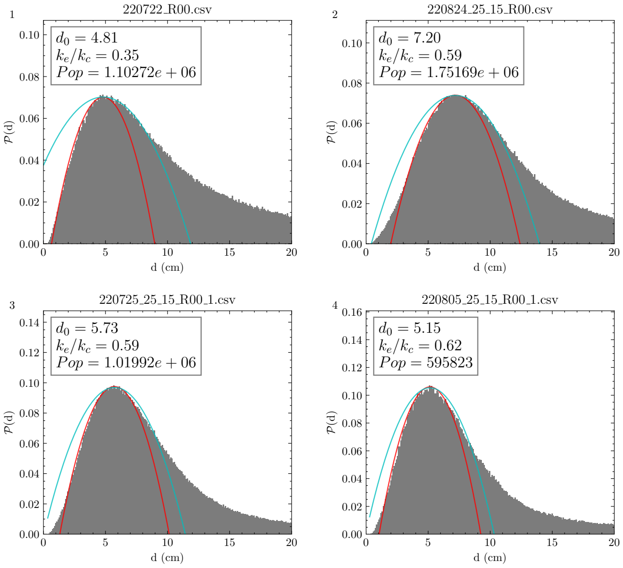

Supplementary Figure 6: Probability distributions of distances (d) between fish that share an edge of their voronoi cell for four separate replicate experiments (1-4) with 25 fish in the largest arena ($R = 45.44$cm). Distributions are fit with two parabolas centered around the most occurring value (d_0). The ratio of the coefficients for the parabolas (k_e/k_c) and population number of data points (Pop) in each probability distribution is included.

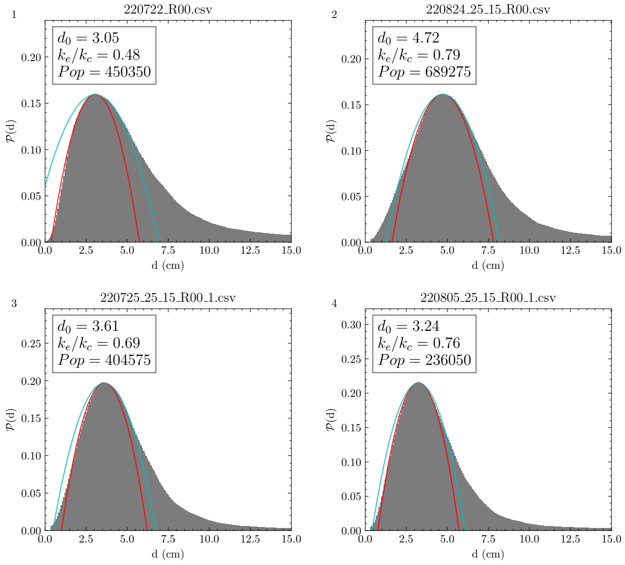

Supplementary Figure 7: Probability distributions of distances (d) between a fish and its closest neighbor that shares an edge of their voronoi cell for four separate replicate experiments (1-4) with 25 fish in the largest arena ($R = 45.44$cm). Distributions are fit with two parabolas centered around the most occurring value (d_0). The ratio of the coefficients for the parabolas (k_e/k_c) and population number of data points (Pop) in each probability distribution is included.
